# Development of Budesonide Loaded Biopolymer Based Dry Powder Inhaler: Optimization, *In Vitro* Deposition, and Cytotoxicity Study

**DOI:** 10.1155/2014/795371

**Published:** 2014-06-15

**Authors:** Ashwin J. Mali, Atmaram P. Pawar, Ravindra N. Purohit

**Affiliations:** Department of Pharmaceutics, Poona College of Pharmacy, Bharati Vidyapeeth University, Erandwane, Pune, Maharashtra 411038, India

## Abstract

The progress in the development of DPI technology has boosted the use of sensitive drug molecules for lung diseases. However, delivery of these molecules from conventional DPI to the active site still poses a challenge with respect to deposition efficiency in the lung. At same time, serious systemic side effects of drugs have become a cause for concern. The developed budesonide loaded biopolymer based controlled release DPI had shown maximum *in vitro* lung deposition with least toxicity. The subject of present study, lactose-free budesonide loaded biopolymer based DPI, further corroborates the great potential of antiasthmatic drugs. This technology is expected to revolutionize the approaches towards enhanced therapeutic delivery of prospective drugs.

## 1. Introduction

Corticosteroids have been found to be very effective for the control of mortality rate and approved as a maintenance therapy in asthmatic patients [1, 2]. Budesonide, a corticosteroid used in the first line therapy for coronary obstructive pulmonary disease (COPD), is available in the market as a conventional dry powder inhale (DPI). The optimum dose for budesonide is ranging between 200 *μ*g and 800 *μ*g. This is a potent nonhalogenated corticosteroid having maximum glucocorticoids receptor activity. The hepatic first pass metabolism of budesonide is approximately 90% which is the main reason for its low oral bioavailability of 6–11% having half-life 2-3 h [3]. The high doses of corticosteroids produce serious side effects upon long-term administration. There is need for controlled release budesonide DPI which could be administered through pulmonary route. Such a formulation could reduce the systemic side effects by achieving high local concentration in the lung and improve the patient compliance [4].

Pulmonary drug delivery system is explored as one of the alternative drug delivery systems due to higher surface area (100–140 m^2^), high permeation of lung, avoidance of hepatic first pass metabolism, and noninvasive route for drug administration [5, 6]. It was found to be the most efficient route for treatment of asthma, chronic obstructive pulmonary disease, and cystic fibrosis and now it is being explored for systemic administration of various categories of drug [7]. Drugs used for cancer, diabetes, and migraine could be efficiently administered by this route. Furthermore peptides, proteins, and genes can be administered through this route as these are stable in the dry form [8].

Conventionally dry powder inhalers (DPIs) are prepared by micronization methods which are often blends of fine drug particles and lactose (carrier) where drug particles are expected to adhere to the carrier surface. The particle morphology, density, and composition cannot be controlled during micronization process which seems to influence cohesive, surface, and electrostatic properties of conventional DPI [9, 10]. This shows only 30% of drug deposition in the lung out of total drug concentration of present conventional dosage forms. This increases the number of doses and frequency of administration. To overcome these problems pharmaceutically improved delivery of dry powder inhaler formulation should be achieved for efficacious drug delivery to achieve local effects in the asthma and COPD which prominently comprises the larger airways region of the lung. Moreover, most of the DPI formulations rely on lactose monohydrate as a carrier where lactose has major drawbacks such as presence of transmissible spongiform encephalopathy and endotoxins obtained from bovine source. Also it cannot be used in the compounds with the reducing sugar such as proteins, peptides, budesonide, and formoterol [11].

The desired performance of dry powder inhaler (DPI) was indicated by its fine particle fraction (FPF) and emitted dose (ED) which in turn mainly depends upon the particle mass median aerodynamic diameter (MMAD). To achieve maximum deposition in the lung, particles exhibiting MMAD ranging from 1 to 5 *μ*m were required [12–14].

Most of the research work and patents came out with various novel systems to achieve required range of MMAD that includes nanoparticles, microspheres, solid-lipid nanoparticles, liposomes, and porous particles. Particle penetration and deposition in the lungs depend on the aerodynamic behavior of particle which changes the particle velocity and direction. Thus particle trajectories depend upon particle dynamics which were governed by the particle density, size, shape, surface nature, and charge of particles [15, 16].

Hickey et al. observed that the static, bulk, and solid state property of lactose in DPI was responsible for better aerodynamic behavior and respiratory deposition [17]. Recently, Divey et al. achieved good lung deposition for DPI containing electrostatically driven hybrid nanoparticles [18]. Telko et al. also studied the effect of triboelectrification on the cohesive and noncohesive types of DPI [19].

Sodium alginate and chitosan, two naturally occurring polymers, were used widely in the formulation development due to their unique properties such as biocompatibility, biodegradability, and forms complexation with polyelectrolyte ions (CaCl_2_) which were attractive to many researchers to formulate carriers like nanoparticles and microparticles with controlled drug release [20–25]. Pluronic F-68 is an amphiphilic synthetic polymer containing hydrophilic poly(ethylene oxide) (PEO) blocks and hydrophobic poly(propylene oxide) (PPO) blocks arranged in triblock structure which has unique property in the encapsulation of drug moiety in the delivery system [7].

Notably, there were no pulmonary formulations present in the market prepared by biopolymer based controlled drug release which would be beneficial to produce local effect with reduced systemic side effect and overcome lung deposition obstacles with improved local inhalation therapy. To further advance the therapeutic utility of budesonide, the present investigation deals with development of hydrophobic budesonide loaded biopolymer (sodium alginate, chitosan, and pluronic F-68) based controlled release microparticulate dry powder inhaler (DPI) with desired physical characteristics and aerosolization in order to improve aerodynamic behavior and lung deposition. The microparticles were prepared by controlled pregelation of sodium alginate solution containing pluronic F-68 followed by polycationic (chitosan) cross-linking technique and 3^2^ factorial design adapted to optimize the amount of chitosan and calcium chloride [26]. The formulations were lyophilized using mannitol as a cryoprotectant to get stable formulations and evaluated in terms of respirable fraction using twin stage impinger (TSI) and powder properties. The optimized formulation was subjected to mass median aerodynamic diameter and fine particle fraction along with static properties on particle dynamics and fluidization evaluation using Andersen cascade impactor (ACI) in comparison with commercial DPI. Further,* in vitro* cell viability against alveolar epithelial cancer cell line A549 was studied to prove the safety of formulation.

## 2. Experiment 

### 2.1. Materials and Method

Budesonide was obtained from Lupin Ltd., Pune, India. Sodium alginate (medium viscosity, 3500 cps) and dialysis bag with a 12,000 molecular weight cutoff was purchased from Sigma Aldrich Chemicals Private Ltd. (Bangalore, India). Budesonide dry powder inhalation commercial product was purchased from local market. Deacetylated chitosan (deacetylation degree 37.08, molecular weight 50 kDa) was obtained from Marine Chemicals, Cochin, India. Pluronic F-68 was provided by Cipla Pharmaceuticals (Mumbai, India). Acetone, potassium dihydrogen phosphate, sodium dihydrogen phosphate, calcium chloride, and all the solvents used in the study were obtained from Merck Ltd. (Mumbai, India).

### 2.2. Fabrication of Budesonide DPI

Budesonide DPI was prepared by the principle involving cation induced controlled gelification of alginate, method reported by Rajaonarivony et al., with slight modifications [27]. Acetone solution (10 mL) of drug (25 mg) and pluronic F-68 (100 mg) was added to the sodium alginate (0.063% w/v) solution under magnetic stirring at 250 rpm, to which optimized 4 mL of calcium chloride (10 mM) solution was added dropwise for 15 min followed by 1 mL of optimized chitosan (2 mg) solution added; stirring was continued for 24 hours until evaporation of organic solvent was completed. The obtained microparticles suspension was subjected to lyophilized using mannitol (2.5% w/v) as a cryoprotectant to get budesonide DPI.

### 2.3. Experimental Design

Process parameters were optimized based on the preliminary data by applying the 3^2^ factorial designs for formulated DPI. The response surfaces of the obtained results were plotted. The coded values are listed in Table 1. The obtained data was analyzed by the results observed from the multiple regression analysis using Design Expert 8.0.6.1 software (Stat-Ease Inc., USA).

The following equation was obtained:

(1) $$\begin{array}{c} Y=\beta_{0}+\beta_{1}X_{1}+\beta_{2}X_{2}+\beta_{11}X_{1}X_{1}+\beta_{22}X_{2}X_{2}+\beta_{12}X_{1}X_{2}, \end{array}$$

where *Y* is the measured response, *X* is the levels of factors, *β* is the regression coefficient, and *X*_1_ and *X*_2_ indicate the amount of calcium chloride and chitosan.

### 2.4. Characterization of Budesonide DPI

#### 2.4.1. Particle Size Analysis of Suspension and Lyophilized Formulation

The mean particle size was determined by laser diffraction technique using Malvern 2000 SM (Malvern Instruments, Malvern, UK) which allows sample measurement in the range of 0.05–20,000 *μ*m. Analysis was carried out at room temperature keeping angle of detection 90°. The mean particle size was expressed in terms of D (0.9), that is, size of the 90% of the particle. The data presented are mean values of three independent samples produced under identical production conditions.

The particle size of the prepared lyophilized formulation was checked by laser diffraction technique using Malvern 2000 SM (Malvern Instruments, Malvern, UK) with the help of dry assembly.

#### 2.4.2. Entrapment Efficiency Analysis

The amount of drug entrapped in the formulations was calculated by estimating the amount of unentrapped drug by centrifugation at 25,000 rpm for 30 min. The obtained supernatant was assayed spectrophotometrically at 246 nm for free drug content. In this process the percent entrapment efficiency (EE) was calculated as the percentage of drug entrapped in the final dosage form to its initial concentration. The %EE was calculated using

(2) %EE=((Initial  drug  concentration)−1(Total  drug  concentration  − Drug  concentration  in  supernatant) × (Initial  drug  concentration)−1)×100.

#### 2.4.3. Flow Properties of Formulated DPI

The fixed height cone method was used to check the flow property of the formulations and commercial DPI. A glass funnel with 5 mm internal diameter was fixed at height of 2.5 cm over the flat surface. The gentle flowing of the powder through the funnel was carried out. The diameter of the powder cone formed was measured. The angle of repose was calculated by the following equation:

(3) $$\begin{array}{c} \text{Tan }\theta =\frac{\text{height}}{\text{radius}}. \end{array}$$

The tapped and untapped densities were evaluated using a small graduated tube with a defined volume size into which the known weight of the powders was added. Bulk density is determined by dividing the mass of the powder by the volume. Tapped volume is calculated by using a tap density tester (Electrolab, tap density tester, USP) following 100 taps. Tapped density is determined by dividing the mass of powder by volume. Carr's index (Ci) is calculated using the values of bulk and tapped density:

(4) $$\begin{array}{c} \text{Ci}=\frac{\left(\text{tapped}\;\text{density}-\text{bulk}\;\text{density}\right)}{\text{tapped}\;\text{density}}\times 100. \end{array}$$

Hausner ratio defines the flowability of powder mixture. The value indicates the ratio of bulk and tapped density:

(5) $$\begin{array}{c} \text{Hausner}\;\text{ratio}=\frac{\text{bulk}\;\text{density}}{\text{tapped}\;\text{density}}. \end{array}$$

Carr's index, Hausner's ratio, and percentage porosity are the tools used to quantify flow properties of powders. The Hausner ratio is <1.25 and Carr's index is in the range of 5–15%.

Percent porosity (*ε*) is used to determine compressibility of powder which is the degree of volume reduction due to an applied pressure which is the measurement of porosity changes during compaction and is calculated using the following formula:

(6) $$\begin{array}{c} E=1-\left(\frac{\text{Pb}}{\text{Pt}}\right)\times 100, \end{array}$$

where Pb and Pt are bulk and taped density of DPI.

#### 2.4.4. *In Vitro* Deposition Study Using Twin Stage Impinger

Rotahaler was used as the delivery device for determinations using twin stage impinger (TSI), Andersen cascade impactor (ACI), and dosage unit sampling apparatus (DUSA). The obtained 25 mg of powder equivalent to 200 *μ*g budesonide was encapsulated in hydroxyl propryl methyl cellulose (HPMC) stick-free capsule ≠3. Initially respirable fraction of optimized budesonide and commercial DPI was determined by TSI (Model number WP-SSGI-0289, Westech Instruments, UK) after aerosolization at 60 ± 5 L/min for 5 sec with 7 mL and 30 mL of phosphate buffer saline (PBS pH 7.4) in stages 1 and 2 of the impinger, respectively. Each stage was rinsed with PBS and drug content was determined by the UV spectrophotometry method after appropriate dilution. Rotahaler with filled capsule to be tested was placed into a rubber mouthpiece attached to the throat of the TSI and the pump was switched on. The pump was operated so as to get the flow rate of 60 ± 5 L/min. The capsule was released by operating the inhalation device and the pump was allowed to run for another 5 seconds which allowed the aspiration of 5 L of air in the apparatus, as recommended by the European Pharmacopoeia (2000). Each section (inhaler, capsule shell, stages 1 and 2) was rinsed with PBS pH 7.4. The rinsed buffer was collected and diluted to an appropriate volume. The budesonide content was determined by UV spectrophotometer at 246 nm (Jasco-v-530). The formulation having the highest respirable fraction was chosen for further deposition studies using an ACI [28–30].

#### 2.4.5. Zeta Potential Analysis

The finalised DPI formulation was checked for the charge assessment. The zeta potential of the formulated DPI was measured by the laser Doppler electrophoretic mobility measurement using Zetasizer 300 HSA (Malvern Instruments Ltd., UK) at temperature of 25°C.

#### 2.4.6. Transmission Electron Microscopy

Transmission electron micrograph (TEM) was obtained for budesonide DPI using a JEOL 1200 EXII TEM. Initially, carbon-coated grids were floated on a droplet of the formulation on a flexible plastic film (Parafilm) to permit the adsorption of the particles onto the grid. After that, the grid was blotted with a filter paper and air-dried for 1 h. Obtained data was used to analyze the size and morphological data of formulated DPI.

#### 2.4.7. Scanning Electron Microscopy

Crystal characteristic of the final formulations was studied by scanning electron microscopy (SEM). Samples were mounted on the aluminum stub and coated with a thin gold-palladium layer by Auto Fine Coater (JEOL, JEC-1600, Tokyo, Japan) and analyzed with a scanning electron microscope (JEOL, JSM-6360A, Tokyo, Japan) operated at an 10 kV acceleration voltage.

#### 2.4.8. Fourier Transform-Infrared Spectroscopy

IR spectra were recorded from 4,000 to 400 cm^−1^ with a Fourier transform-infrared spectrometer (FTIR-8400; Shimadzu Corporation, Kyoto, Japan) equipped with a diffuse reflectance accessory (DRS-8000; Shimadzu Corporation, Japan) and a data station to confirm drug entrapment in the polymer. About 2-3 mg samples were prepared by processing compressed KBR discs.

#### 2.4.9. Differential Scanning Calorimetry

The differential scanning calorimetry (DSC) thermograms of formulated DPI were obtained using DSC 821e (Mettler-Toledo, Greifensee, Switzerland). Indium standards were used to calibrate the temperature and enthalpy scale. Samples were (5–10 mg) heated in hermetically sealed aluminium pan with a heating rate of 10°C/min over a range of 0–300°C under a nitrogen atmosphere (flow rate 50 mL/min).

#### 2.4.10. Powder X-Ray Diffraction

Powder X-ray diffraction (PXRD) patterns of particles were recorded by X-ray diffractometer (PW 1729; Philips, Almelo, The Netherlands) using Cu K*α* radiation (1.542 A) with a voltage of 30 kV and a current of 30 mA. Samples were scanned from 10° to 30° at 2*θ*.

#### 2.4.11. Release Profiles

The* in vitro* release for budesonide loaded biopolymer based DPI was carried out in phosphate buffer saline (pH 7.4) using dialysis bag diffusion technique. Formulation equivalent to 200 *μ*g of budesonide was added into the dialysis bag (cellulose membrane, mw cutoff 12,000 Da), which was hermetically sealed and immersed into 100 mL of release medium. The entire system was kept at 37 ± 0.5°C with continuous magnetic stirring at 100 rpm/min. At selected time interval, sample was removed and replaced with fresh medium in order to maintain sink conditions. The sample was analyzed by UV spectrophotometry at 246 nm.

#### 2.4.12. *In Vitro* Deposition Study Using ACI

An aerodynamic characteristic of optimized budesonide DPI having minimum particle size, maximum entrapment efficiency, and excellent flow properties was assessed and compared with the commercial DPI (Budecort Rotacpas) by using an eight-stage, nonviable cascade impactor (Westech private instruments, Model Number WP-ACISS-0289). The obtained 25 mg of powder equivalent to 200 *μ*g budesonide was encapsulated in hydroxyl propryl methyl cellulose (HPMC) stick-free capsule ≠3. Rotahaler was used as delivery device. The capsule to be tested was placed in the Rotahaler, which had been fitted into moulded rubber mouthpiece attached to the throat piece of the impactor. Once assembly had been checked and found to be vertical and stable, run was conducted at a flow rate of 60 L/min for 5 sec. The capsule shell was removed from the inhaler device and four more capsules were actuated in the same manner. The test was conducted in triplicate. Cutoff particle aerodynamic diameters at 60 L/min for each stage of the impactor were preseparator (8.6 *μ*m), stage 0 (6.5 *μ*m), stage 1 (4.4 *μ*m), stage 2 (3.3 *μ*m), stage 3 (2.0 *μ*m), stage 4 (1.1 *μ*m), stage 5 (0.54 *μ*m), and stage 6 (0.25 *μ*m). After the completion of dosing, different plates were collected; they were washed with 10 mL of acetonitrile: phosphate buffer saline (pH 3.2). The dispersion was sonicated in a bath-type sonicator for 15 min. Then the solution was centrifuged at 25,000 rpm for 30 min and the amount of budesonide in the supernatant was determined using a high performance liquid chromatography (HPLC) assay method. The deposition of formulated and commercial DPI on each stage of the impactor was determined. MMAD and GSD were calculated from the deposition data using the MMAD calculator for Anderson apparatus [13, 31, 32].

The HPLC system specifications were as follows: pump, PU-1580 (JASCO, Japan); injector, Autosampler (AS-1555; JASCO); column, Phenomenex C18, 250 × 4.6 mm, 5 *μ*m (Thermo Electron Corporation, USA); and detector, UV/visible (UV-1575; JASCO). Data acquisition and analysis were carried out using Borwin/HSS 2000 software (LG 1580-04; JASCO). The mobile phase was a mixture of acetonitrile: phosphate buffer saline pH 3.2 (34 : 66 v/v). The column temperature and flow rate were 40°C and 1.5 mL/min and the wavelength was 240 nm.

### 2.5. Cell Viability Assay

*In vitro* cell viability was evaluated for formulated budesonide DPI against alveolar epithelial cancer cell line A549 (obtained from NCCS, Pune, Maharashtra, India) using MTT assay. The results were compared with free budesonide and formulation excipients. The cells were cultured in DMEM/F12 medium and supplemented with 10% v/v fetal bovine serum and 2 mM L-glutamine. The medium maintained humidity atmosphere less than 5% carbon dioxide at 37°C. Trypsin-EDTA solution was used for subculturing and cell isolation.

The cells were harvested on the fourth day of subculture. The cells were seeded at the density of 5 × 10^3^ cells per well and grown in 96-well tissue culture plates in a final volume of 150 *μ*L in humidified atmosphere for 48 hours. Each formulation was dispersed in water and tested in varying budesonide concentration over the range of 15 *μ*M to 1000 *μ*M. After 24 hr of incubation, 10 *μ*L of MTT labeling agent (5 mg/mL in PBS) was added and incubated for further 4 h in humidified condition. After incubation, 100 *μ*L of solubilizing solution (10% SDS in 0.01 M HCl) was added to each well. The plate was incubated overnight. The optical density was measured at 570 nm with a reference wavelength at 630 nm using an ELISA reader. The cell viability was calculated using following equation:

(7) $$\begin{array}{c} \text{Viability}\;\left(\%\right)=\frac{A_{\text{test}}}{A_{\text{control}}}\times 100, \end{array}$$

where *A*_test_ is the absorbance of the test solutions and *A*_control_ is the absorbance of control (PBS).

## 3. Result and Discussion 

Budesonide is a potent corticosteroid used in the first line therapy for coronary obstructive pulmonary diseases (COPD). The low oral bioavailability of budesonide due to hepatic metabolism and short half-life continues to be highlighted as a major challenge in developing formulations for clinical efficacy. However, budesonide is available in the market as a conventional dry powder inhaler (DPI) which shows only 30% of drug deposition in the lung out of total drug concentration. Besides, high/frequent dose is needed to achieve optimum therapeutic efficacy, which often causes severe side effects.

In present study we fabricated budesonide loaded biopolymer carriers based DPI via controlled gelation of sodium alginate where calcium ions react with guluronic acid units of the sodium alginate to form the negative charged calcium alginate polyelectrolyte complex in which drug molecules were entrapped followed by enveloping with chitosan in order to overcome commercial DPI problems. In preliminary study, the amount of calcium chloride and chitosan showed pronounced effect on biopolymer DPI [21, 32]. To investigate the effect of independent variables such as calcium chloride (*X*_1_) and chitosan (*X*_2_) on the dependent variables such as particle size (*Y*_1_), entrapment efficiency (*Y*_2_), bulk density (*Y*_3_), and Carr's index (*Y*_4_) which are major contributing factors for the lung deposition were optimized by using 3^2^ factorial design [26, 28].

### 3.1. Characterization of Budesonide Loaded Biopolymer Based DPI

#### 3.1.1. Particle Size

Significant particle size variations were observed with different concentration of calcium chloride and chitosan. The particle size distribution for formulations F1 to F9 showed values in the range of 1.192 ± 0.03 *μ*m to 3.424 ± 0.04 *μ*m as listed in Table 2. For the commercial DPI particle size was 1.521 ± 0.04 *μ*m. The multiple regression analysis for the mean particle size of factorial batches revealed the fair fit (*R* ^2^ = 0.460). The positive coefficient for both independent variables influencing the size of the particle was given by the following equation:

(8) Y1=7.368+3.513X1+1.136X2+0.346X1X1−0.257X2X2+0.152X1X2.

**Figure 1:**
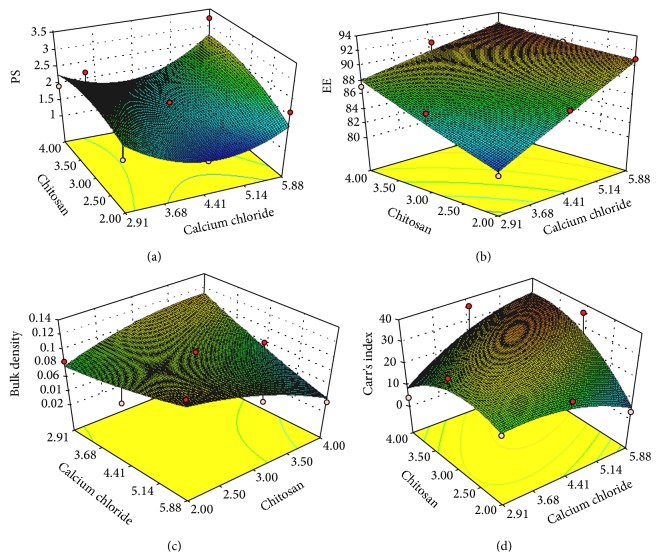
Response surface plots of (a) particle size, (b) entrapment efficiency, (c) bulk density, and (d) Carr's index.

As per the 3^2^ factorial design surface response graph (Figure 1(a)) and polynomial equation (2), the concentration of calcium chloride (*X*_1_) was found to influence change in the particle size. The calcium ions react with glucuronic acid molecules present in sodium alginate, leading to formation of compact polyelectrolyte crosslinked structures. The increased concentration of calcium chloride results in gelation and crosslinking of the biopolymer which was responsible for increase in particle size. Similarly, the chitosan showed the same response as that of calcium chloride in the particle size. The particle size was increased with increasing chitosan (*X*_2_) concentration which may be due to interaction of cationic chitosan polymer with sodium alginate and formation of thick layer coating of excessive chitosan around the particles [21, 28, 33, 34].

#### 3.1.2. Entrapment Efficiency

The effect of independent variables *X*_1_ and *X*_2_ on the percent entrapment efficiency of drug for all the formulations was observed. EE was in the range of 80.68 ± 2.68% to 92.64 ± 2.12% as listed in Table 2. The multiple regression analysis for the EE as per the factorial designs revealed the good fit (*R* ^2^ = 0.943) with the following equation:

(9) Y2=60.018+5.323X1+5.303X2−0.0362X1X1+0.116X2X2−0.884X1X2.

As per the 3^2^ factorial design response surface graph (Figure 1(b)) and polynomial equation (3), EE was mainly governed by concentration of calcium chloride (*X*_1_) which results in lower entrapment in the initial formulations due to weak gel strength and increased entrapment as the concentration of calcium chloride was increased [21]. The formulated DPI showed less entrapment due to “calcium saturation phenomenon” as compared to F8 and F9 [35]. The higher concentration of chitosan (*X*_2_) was also responsible for increasing the EE of drug as it has a film forming property encapsulating the inner core of the particle [33, 36]. The use of triblock polymer showed positive effect in case of EE which might be due to its self-assembling property in aqueous environment with hydrophobic core and intercalation of hydrophilic chain with alginate chitosan complex [37].

#### 3.1.3. Bulk Density and Carr's Index of the Budesonide DPI

Bulk density of all formulations was in the range of 0.037 ± 0.06 g/cm^3^ to 0.123 ± 0.03 g/cm^3^ as listed in Table 3. The multiple regression analysis for the bulk density as per the factorial designs revealed the good fit (*R* ^2^ = 0.823) with the following equation:

(10) Y3=0.147+0.033X1+0.125X2+1.696X1X1−9.333X2X2−0.018X1X2.

As per the 3^2^ factorial design response surface graph (Figure 1(c)) and polynomial equation (4), the formulation interaction term *X*_1_ *X*_1_ has positive influence on the bulk density than the interaction term *X*_1_ *X*_2_ as indicated in (10). The calcium chloride and chitosan demonstrated positive impact on the density of formulations. Insignificant changes in the densities were observed with change in concentrations of calcium chloride and chitosan. The incorporation of materials like chitosan, calcium chloride, and sodium alginate reduced the density with least variations which was helpful to improve flow properties of the formulated DPI.

The Carr index of all the formulations was in the range of 4.65 ± 0.01% to 47.88 ± 0.07% as listed in Table 3, resulting in fair fit (*R* ^2^ = 0.629). The following equation was observed:

(11) Y4=−21.017+2.596X1+24.523X2−2.347X1X1−8.353X2X2+6.639X1X2.

As per the 3^2^ factorial response surface graph (Figure 1(d)) and polynomial equation (5), positive influence of *X*_2_ was seen on the flow property of formulated DPI. Chitosan may be helpful in getting spherical particles by forming thin coat around the formulated DPI which in turn may help to increase the flow property of formulated DPI [21]. From the polynomial equation, response parameters such as EE and density showed good fit which were more significant due to controlled gelation of sodium alginate.

#### 3.1.4. Flow Properties

The aerosolization efficiency of the formulated DPI was governed by the flow properties. The angle of repose, Carr's index, and Hausner's ratio for F1 to F9 formulations were in the range of 24 ± 0.09° to 28 ± 0.02°, 4.05 ± 0.01% to 47.88 ± 0.07%, and 0.52 ± 0.08 to 0.95 ± 0.08 as compared to 24 ± 0.07°, 19.48 ± 0.03%, and 0.80 ± 0.04 for commercial DPI, respectively, as listed in Table 3. The better angle of repose and Carr's index were observed for optimized budesonide DPI as compared to the commercial DPI and remaining formulations. The percentage porosity of all the formulations ranges from 10 ± 0.05% to 48 ± 0.04% as compared to the 20 ± 0.04% of the commercial product.

#### 3.1.5. *In Vitro* Deposition Study Using Twin Stage Impinger

The amount of drug deposited in the second stage of impinger (effective cutoff diameter <6.4 *μ*m) was considered as fine particle dose (FPD). The recovered dose (RD) is the amount of drug present in stage 1 and stage 2 of the impinger, inhaler device, and capsule shell. Respirable fraction (RF) was the ratio of FPD to RD and was expressed in percentage. RF for all the formulations ranges from 30.40 ± 0.03% to 43.10 ± 0.02%. As per the obtained results depicted in Table 2, FPD for all the formulations ranges from 29.75 ± 0.02 *μ*g to 60.09 ± 0.01 *μ*g and RD was in the range of 66.06 ± 0.03 *μ*g to 139.41 ± 0.03 *μ*g. The respirable fraction for F7 was 43.10 ± 0.02% as compared to 22.39 ± 0.05% for commercial DPI. The high FPD of F7 can be attributed to the collective effect of uniform spherical nature, lack of surface van der Waals forces, less bulk density, and good flow property of formulated DPI.

Considering the results of 3^2^ factorial design, the F7 batch showed optimum entrapment efficiency, fine particle dose, respirable fraction, angle of repose, bulk density, tapped density, Carr's index, Hausner's ratio, and percentage porosity which were subjected to further evaluation. The optimized batch F7 showed increased particle size (3.059 ± 0.03 *μ*m) after lyophilization which may be due to aggregation during lyophilization process. The final composition of formulated DPI with respect drug to powder ratio was 1 : 30 mg.

#### 3.1.6. Zeta Potential

**Figure 2:**
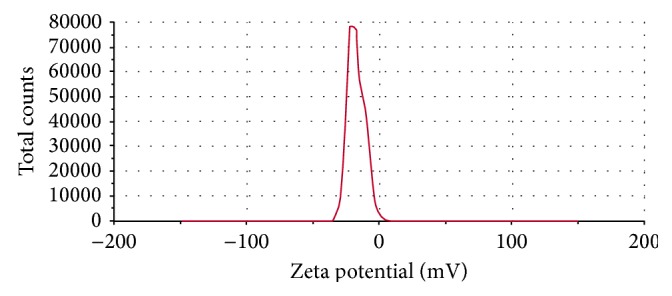
Zeta potential of formulated DPI.

The final formulation has shown −17.5 mV of surface charge (Figure 2). This has resulted from higher concentration of calcium chloride than the chitosan in the final formulation where calcium ions cooperatively bind the alginates molecules preventing chitosan from forming the coat around the alginate molecules. Also it may happen due to inadequate deacylation of chitosan used in the final formulation where stretching of deacetylated chains was not fully carried out due to electrostatic repulsion between the NH_3_ groups that may yield irregular and nonuniform coating of the chitosan resulting in negative charge of the particles [21, 33, 36, 37]. The charge on the human respiratory tract is negative due to presence of mucin [38]. As per the charge theory, negatively charged particles are more responsible for repulsion in between the particles. Therefore, negative charge on the respiratory tract and formulated DPI was responsible for more prominent repulsive forces and was responsible for increasing the time of flight of the budesonide DPI which leads to increasing the deposition of drug in the larger airway region of the lung.

#### 3.1.7. Transmission Electron Microscopy

**Figure 3:**
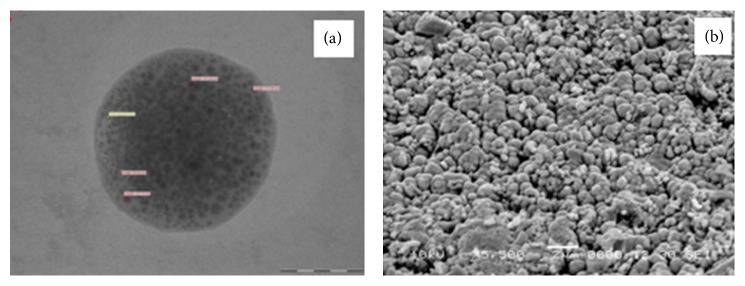
(a) TEM image and (b) SEM image of formulated DPI.

As observed from the TEM depicted in Figure 3(a) the image clearly indicates the presence of drug particles encapsulated in the microparticles of formulated DPI. Observed particles have uniform spherical nature.

#### 3.1.8. Scanning Electron Microscopy

The surface nature and morphology of the formulated DPI were verified by SEM technique. Optimized budesonide DPI as evident from the photograph depicted more uniform spherical particles with smooth surface as shown in Figure 3(b). The SEM image also significantly specifies the uniformity of size and least amount of fines in the formulated DPI at specific range of magnification.

#### 3.1.9. Fourier Transform-Infrared Spectroscopy

**Figure 4:**
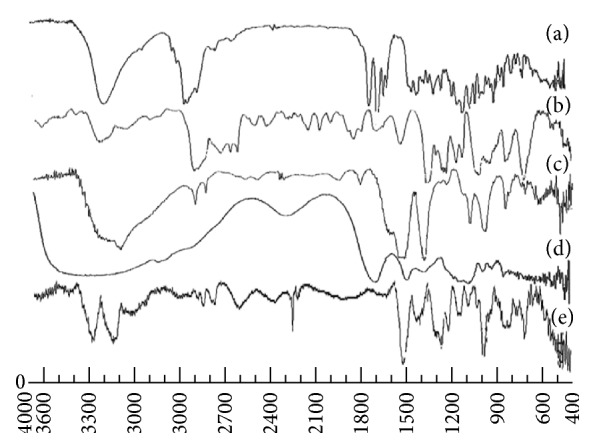
The FTIR of (a) budesonide, (b) formulated DPI, (c) chitosan, (d) sodium alginate, and (e) pluronic F-68.

Potential intermolecular interactions between the polymers and drugs were analyzed by the FTIR spectra (Figure 4). Budesonide showed peaks at 3499 cm^−1^, 2956 cm^−1^, 1722 cm^−1^, and 1690 cm^−1^ due to O–H stretching, C–H stretching, and C=O stretching. The characteristic peaks of sodium alginate were observed at 3357 cm^−1^, 1601 to 1407 cm^−1^, and 1029 cm^−1^ due to hydroxyl group, COO^−^ group, symmetric and asymmetric stretching vibrations, and C–O–C group stretching vibrations, respectively. Chitosan spectra showed peaks at 3414 cm^−1^, 1538 cm^−1^, 1402 cm^−1^, and 1101 cm^−1^ due to presence of N–H stretching of amine group and presence of secondary hydroxyl group. Pluronic F-68 showed functional group peak at 1154.19 cm^−1^. However, in the final spectrum of formulation, budesonide showed minor shifting of peaks to 3487 cm^−1^, 2971 cm^−1^, 1705 cm^−1^, and 1638 cm^−1^ for O–H stretching, C–H stretching, and C=O stretching. Minor shifting in the peaks of sodium alginate was observed at 3987 cm^−1^, 1638 cm^−1^ to 1562 cm^−1^, and 963 cm^−1^ for OH, COO^−^, and C–O–C groups, respectively. Furthermore, in chitosan, shifting of NH_2_ group, amide group, and N–H stretching and hydroxyl group was carried out to 3487 cm^−1^, 1467 cm^−1^, 1459 cm^−1^, and 1136 cm^−1^, respectively. This shifting of functional groups was attributed to the formation of hydrogen bonding and conversion to amorphous form [4, 39].

#### 3.1.10. Differential Scanning Calorimetry

**Figure 5:**
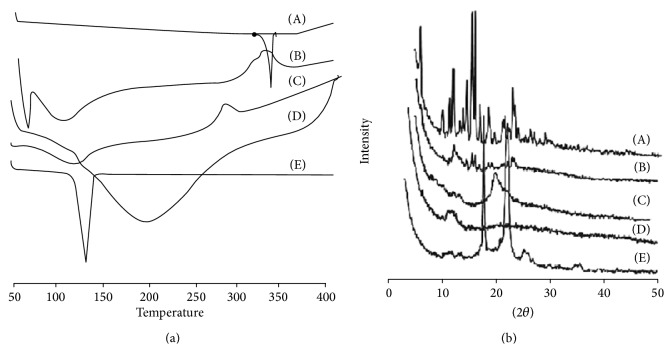
(a) DSC plots and (b) PXRD plots of (A) budesonide, (B) formulated DPI, (C) chitosan, (D) sodium alginate, and (E) pluronic F-68.

In Figure 5(a), DSC scan of budesonide showed sharp endothermic peak at 260°C due to melting transition point of drug. Chitosan exhibited endothermic peak at 104.93°C and exothermic peak at 265.30°C due to the melting and consequently degradation of polymer at higher temperature. DSC scan of sodium alginate showed broad endothermic peak at 105.69°C due to evaporation of water content. Pluronic F-68 showed endothermic peak at 35.20°C due to the melting of polymer. In the physical mixture endothermic peaks at 49.98°C, 118.70°C, and 309.34°C were observed. These peaks may be attributed to loss of water, interaction between the polymers, and melting of polymers at respective temperatures. The final formulation showed the endothermic peak at 81.05°C and exothermic peak at 260.29°C. These peaks mainly represent melting of polymer and degradation of system at higher temperature. The absence of endothermic peak of budesonide in the entire spectrum of formulation pointed out complete entrapment and reduction of drug crystallinity in polymer matrix [36].

#### 3.1.11. Powder X-Ray Diffraction

Peaks with reduced intensity were observed at the formulated DPI as compared to the pure drug. The PXRD diffraction data of pure drug revealed characteristic peaks at 2*θ* of 6.2°, 12.2°, 15.6°, 16.1°, and 23° representing high crystalline nature (Figure 5(b)). Complete disappearance of high intensity peaks in the lyophilized powder was due to formation of complex in the polymer matrix. The intermolecular interaction between polymer matrix and drug molecules results in the molecular complex which was responsible for less intensity peaks.

#### 3.1.12. Release Profile

**Figure 6:**
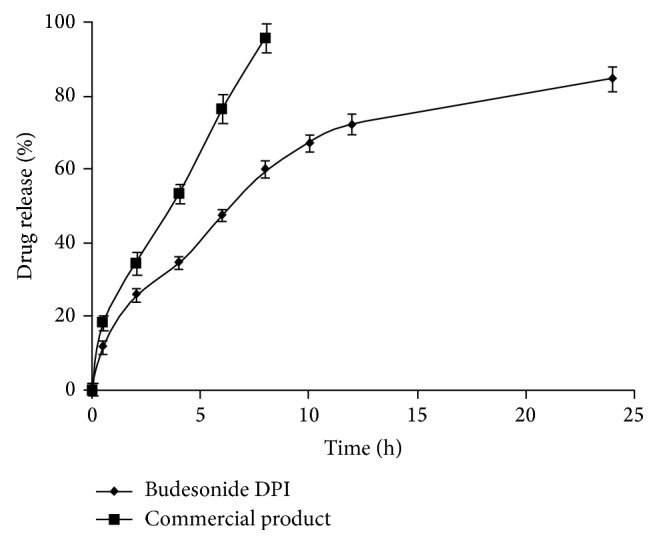
*In vitro* drug release profile of formulated budesonide and commercial DPI. Data are presented as mean ± SD, *n* = 3.

*In vitro *drug release profiles of budesonide from DPI were carried out by dialysis technique using diffusion bag. The release studies were carried out in PBS (pH 7.4) at 37°C. As shown in Figure 6, the rapid release of budesonide from commercial DPI was observed, nearly 100% in 8 h due to rapid diffusion of budesonide in PBS. The obtained DPI showed a biphasic release pattern with initial burst release (25%) within the first 2 h followed by controlled release up to 24 h. The initial burst release may be due to the presence of free drug or adsorption on the surface of the microparticles, while a controlled release could be caused by diffusion of the drug from rigid polymeric chains of gelled biodegradable sodium alginate [40]. The drug entrapped into the inner core compartment stayed firmly inside the microparticles showing a very slow release even at sink conditions with 16% of the initially incorporated drug still being associated with the microparticles even after 24 h. The controlled release reflects the longer retention of drug in the lung which reduces the exhalation and systemic toxicity of budesonide.

#### 3.1.13. *In Vitro* Deposition Study Using Andersen Cascade Impactor

The aerodynamic diameter is the key factor for drug deposition in the lung. The key parameters such as FPF, MMAD, and GSD prominently decided the aerosolization efficiency and deposition of drug in the lungs. According to European Pharmacopeia, the HPLC analytical method and process of extraction were well validated in which budesonide active metabolites peaks were eluted at 17.6 min and 19.2 min in phosphate buffer pH 3.2. The peaks areas of metabolites were used for quantification. The metabolites calibration curve was linear (*y* = 22352*x* + 41669) at a concentration range of 0.001–50 *μ*g/mL. In order to determine the drug deposition in various stages, Rotahaler was connected to the cascade impactor at 60 L/min and drug content was calculated on each stage.

The optimized budesonide DPI showed the MMAD 1.16 ± 0.01 *μ*m as compared to 5.04 ± 0.03 *μ*m for commercial DPI as stated in Table 4. This was observed due to the lower density of formulated budesonide DPI [4, 8]. Particles with MMAD of 1–3 *μ*m are responsible for efficient alveolar deposition. Therefore, the formulated DPI having MMAD 1.16 ± 0.01 *μ*m is expected to deposit prominently in the lower region of lung as compared to the commercial DPI.

The %RF, referred to also as the fine particle fraction of the total dose (FPF), was calculated as the percentage of aerosolized particles that reached the lower seven stages of the impactor (corresponding to aerodynamic diameters below 5.8 *μ*m) or the lower five stages (corresponding to aerodynamic diameters below 3.3 *μ*m) according to the following equation [13, 41]. The FPF is calculated as

(12) %FPF=((Total  particle  mass  recovered)−1(Powder  mass  recovered  from  the  terminal stages  of  impactor) ×(Total  particle  mass  recovered)−1)×100.

The FPF for formulated budesonide DPI was 56.18 ± 0.05%. The commercial DPI has FPF of 22.83 ± 0.06%. The optimized budesonide loaded biopolymer based DPI exhibited one-and-half-fold increase in deposition at the terminal stages of impactor with efficient aerosolization as compared to the commercial DPI. Most of the commercial DPI formulations are blend of micronized drug with larger carrier particles in a specific ratio where particle separation is the most important performance characteristic for effective aerosol generation, but due to the micronization and blending process there is the chance of induction of surface and electrostatic charges on the drug particles [10]. The particle morphology, density, and composition cannot be effectively controlled. Therefore, powder turns to be more cohesive and poorly flowable which mainly affects the particle trajectories and lung deposition at adequate shear force of the inhaled air [42, 43]. In the optimized budesonide DPI, there were least chances of cohesiveness due to bypass of micronization and blending method. The least differences in the bulk and tapped density of the formulated DPI as compared to the commercial DPI were due to the presence of uniformity in the particles which imparts higher fluidization and trajectories in the powder bed and helps in efficient deposition of formulated DPI [44]. Moreover, formulated DPI has shown negative surface charge of −17.5 mV (Figure 2). As per the charge theory, negative surface charge on the respiratory tract and formulated DPI was more prominently responsible for repulsive forces which may increase the time of flight and consequently lung deposition of the budesonide DPI.

### 3.2. Cell Viability Assay

**Figure 7:**
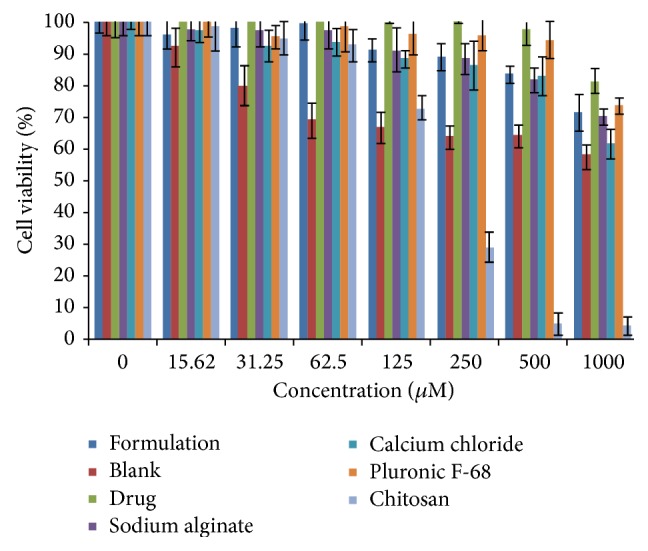
Percentage cell viability against alveolar epithelial cancer cell line A549 of formulated budesonide and blank DPI and its excipients. Data are presented as mean ± SD, *n* = 3.

As the microparticles are intended to provide control release, it is necessary to test for local toxicity of the formulation and its excipients [45]. Therefore,* in vitro* cell viability for optimized budesonide loaded biopolymer based DPI was evaluated against alveolar epithelial cancer cell line A549 using MTT assay and compared with blank formulation, free budesonide, and formulation excipients (Figure 7). At 500 *μ*M concentration, all the tested formulations showed more than 80% cell viability, whereas the blank formulation and chitosan showed 64% and 4.9% cell viability, respectively. However, the concentrations of all the excipients used were less than 500 *μ*M. Even, at 1000 *μ*M concentration, the formulated budesonide DPI showed 71.7% cell viability. The improved cell viability in the formulated DPI due to negative charge of engineered particles and controlled release of the drug from rigid polymeric chains of gelled biodegradable sodium alginate microparticles leads to lower cellular internalization [46]. The results indicated that the formulated biopolymer based DPI was safe up to 1000 *μ*M.

## 4. Conclusion

Formulation of statistically optimized budesonide loaded biopolymer based DPI was carried out by using biocompatible sodium alginate polymer which was useful to enhance the fluidization with increased regional lung deposition. The characteristic of the formulated DPI was predominantly influenced by calcium chloride and chitosan. The optimized biopolymer based DPI results in better* in vitro* lung deposition as compared to the commercial DPI by using TSI and ACI. The study revealed predominant correlations between the flowability, surface charges, and physical properties as compared to particle size for particle dynamics in the respiratory tract. From the results it can be concluded that, for effective particle fluidization and trajectories, along with morphological properties, there was higher probing influence of surface charge of formulated DPI and acts as merit for evaluation of lung deposition.* In vitro* cell viability against alveolar epithelial cancer cell line A549 proved safety of formulation. Further* in vivo *regionallung deposition studyis in the pipeline.

## Figures and Tables

**Table 1:** Factorial design of all formulations.

Formulation	Factorial design	Calcium chloride (mL)	Chitosan (mg)
F1	(−1, −1)	1	2
F2	(−1, 0)	1	3
F3	(−1, +1)	1	4
F4	(0, −1)	2	2
F5	(0, 0)	2	3
F6	(0, +1)	2	4
F7	(+1, −1)	3	2
F8	(+1, −1)	3	3
F9	(+1, +1)	3	4

**Table 2:** Characterization of *in vitro* deposition of formulations by TSI.

Formulation number	*D*[0.9] [*μ*m]^a^	Entrapment efficiency [%]^a^	Recovered dose [*μ*g]^a^	Fine particle dose [*μ*g]^a^	Respirable fraction [*μ*g]^a^
F1	1.761 ± 0.05	80.68 ± 2.68	123.80 ± 0.04	52.15 ± 0.02	42.12 ± 0.02
F2	1.192 ± 0.03	86.43 ± 1.15	116.28 ± 0.02	48.84 ± 0.01	42.00 ± 0.03
F3	2.147 ± 0.03	90.92 ± 2.21	112.15 ± 0.03	44.87 ± 0.04	40.00 ± 0.01
F4	3.204 ± 0.01	85.94 ± 2.12	110.6 ± 0.06	44.24 ± 0.06	40.00 ± 0.05
F5	1.926 ± 0.03	86.66 ± 1.25	130.28 ± 0.04	51.81 ± 0.03	39.76 ± 0.02
F6	3.424 ± 0.04	92.64 ± 2.12	129.41 ± 0.03	47.51 ± 0.02	36.71 ± 0.04
F7	1.937 ± 0.06	87.16 ± 1.11	139.41 ± 0.03	60.09 ± 0.01	43.10 ± 0.02
F8	1.537 ± 0.08	91.39 ± 1.98	97.86 ± 0.01	29.75 ± 0.02	30.40 ± 0.03
F9	3.218 ± 0.09	92.20 ± 2.25	66.06 ± 0.04	30.03 ± 0.03	37.50 ± 0.01
Commercial DPI	—	—	48.31 ± 0.03	10.82 ± 0.03	22.39 ± 0.05

(+1) = higher values and (−1) = lower values.

^a^All the determinations performed in triplicate and values are expressed as mean (values = average ± SD).

**Table 3:** Flowability characteristics of budesonide DPI.

Formulations	Angle of repose^a^ [*θ*]	Bulk density^a^ [g/cm^3^]	Tapped density^a^ [g/cm^3^]	Carr's index^a^ [Ci%]	Hausner ratio^a^	Percentage porosity^a^
F1	26 ± 0.01	0.084 ± 0.04	0.105 ± 0.05	20.00 ± 0.04	0.80 ± 0.09	20 ± 0.04
F2	26 ± 0.07	0.071 ± 0.02	0.123 ± 0.02	42.27 ± 0.02	0.57 ± 0.08	43 ± 0.07
F3	24 ± 0.04	0.123 ± 0.03	0.129 ± 0.04	04.65 ± 0.01	0.95 ± 0.08	10 ± 0.05
F4	27 ± 0.01	0.097 ± 0.02	0.131 ± 0.07	25.95 ± 0.03	0.74 ± 0.01	26 ± 0.02
F5	25 ± 0.02	0.101 ± 0.07	0.124 ± 0.02	18.54 ± 0.05	0.81 ± 0.03	19 ± 0.01
F6	24 ± 0.09	0.073 ± 0.02	0.089 ± 0.01	17.97 ± 0.03	0.80 ± 0.04	18 ± 0.08
F7	25 ± 0.06	0.076 ± 0.08	0.095 ± 0.02	20.00 ± 0.02	0.80 ± 0.10	20 ± 0.03
F8	28 ± 0.02	0.079 ± 0.02	0.124 ± 0.03	36.29 ± 0.03	0.63 ± 0.03	37 ± 0.06
F9	26 ± 0.05	0.037 ± 0.06	0.071 ± 0.02	47.88 ± 0.07	0.52 ± 0.08	48 ± 0.04
Commercial DPI	24 ± 0.07	0.124 ± 0.05	0.154 ± 0.09	19.48 ± 0.03	0.80 ± 0.04	20 ± 0.04

^a^All the determinations performed in triplicate and values are expressed as mean (values = average ± SD).

**Table 4:** Characterization of *in vitro* deposition of final formulated and commercial DPI by ACI.

Formulation	Particle size of dry powder [0.9]^a^	Angle of repose [*θ*]	Bulk density^a^ [g/cm^3^]	Tapped density [g/cm^3^]	Carr's index^a^	Hausner ratio^a^	MMAD^a^ [*μ*m]	GSD^a^ [*μ*m]	FPF^a^ [%]
Optimized DPI (F7)	3.059 ± 0.03	25 ± 0.01	0.076 ± 0.01	0.095 ± 0.02	20.00 ± 0.01	0.80 ± 0.10	1.16 ± 0.01	3.78 ± 0.07	56.18 ± 0.05
Commercial DPI	1.521 ± 0.04	24 ± 0.01	0.124 ± 0.01	0.154 ± 0.01	19.48 ± 0.03	0.80 ± 0.01	5.04 ± 0.03	1.44 ± 0.02	22.83 ± 0.06

^a^All the determinations performed in triplicate and values are expressed as mean (values = average ± SD).
